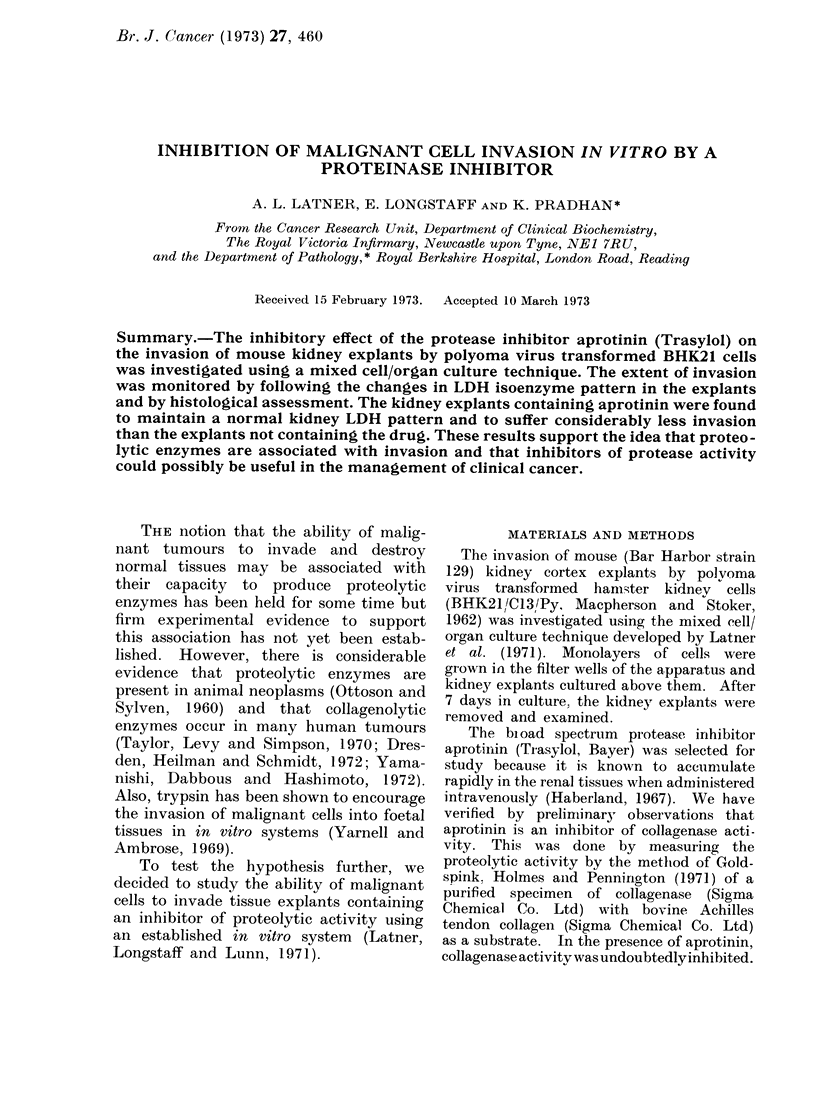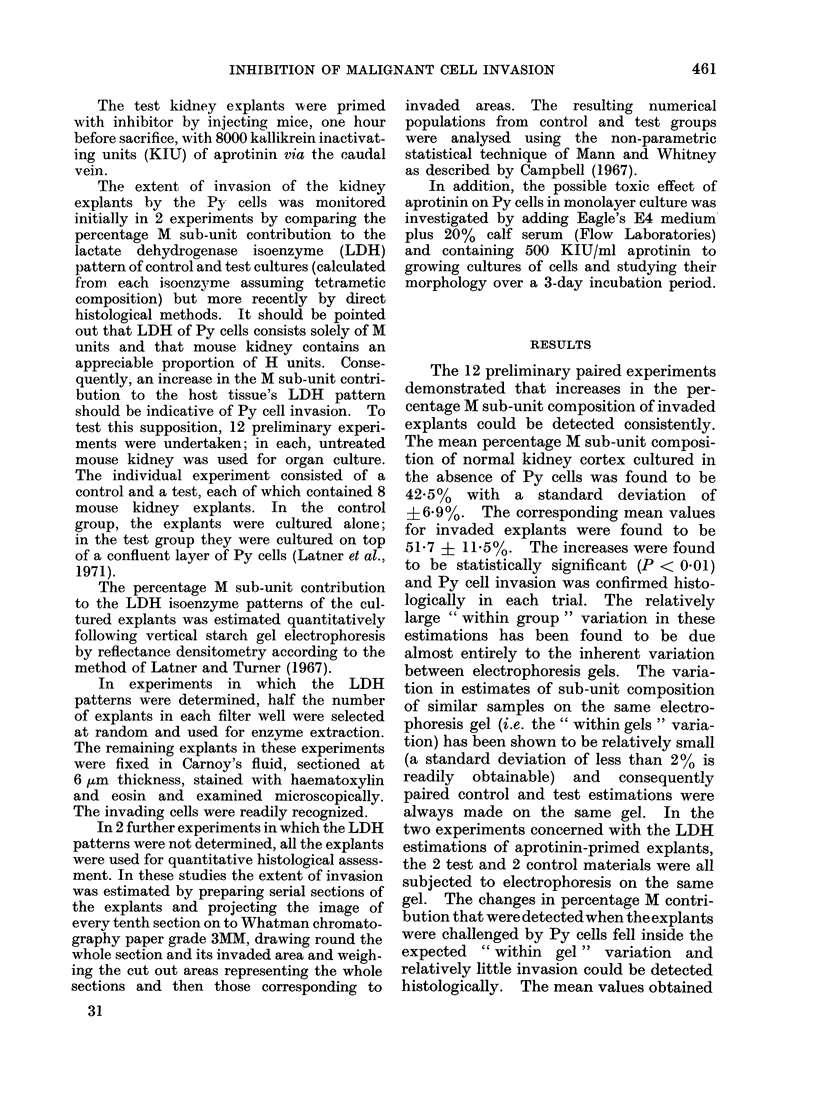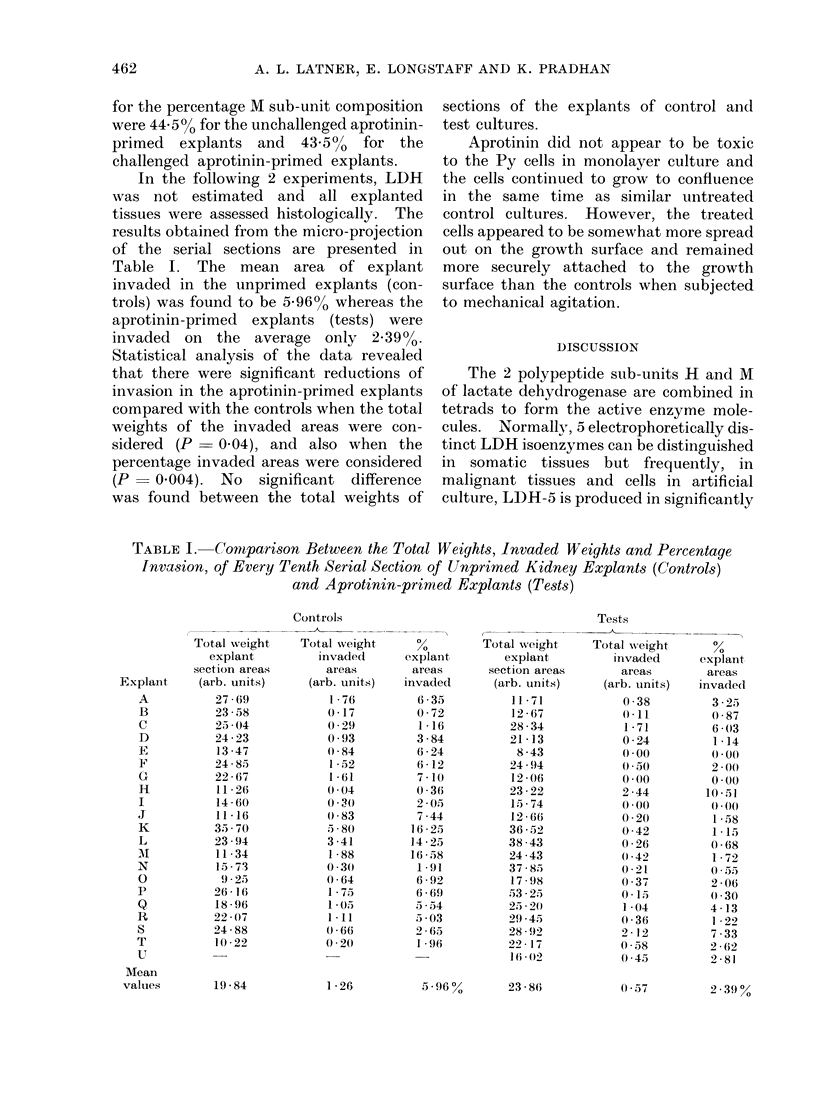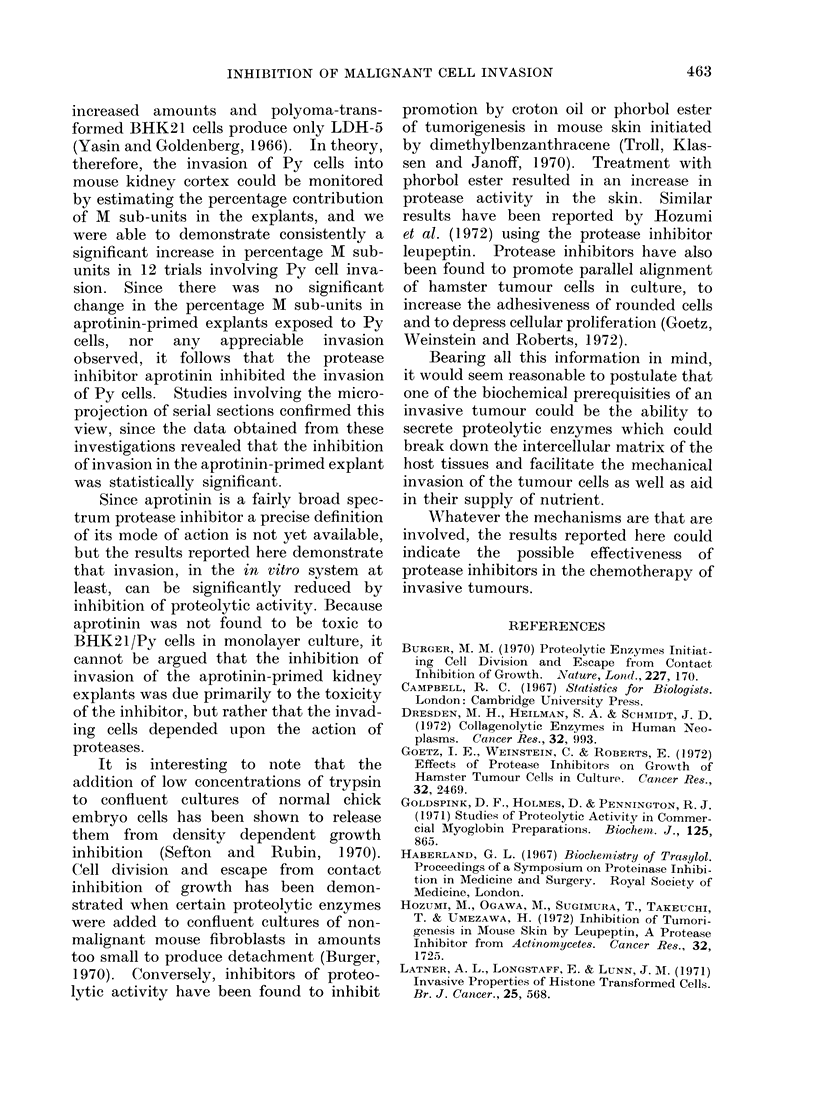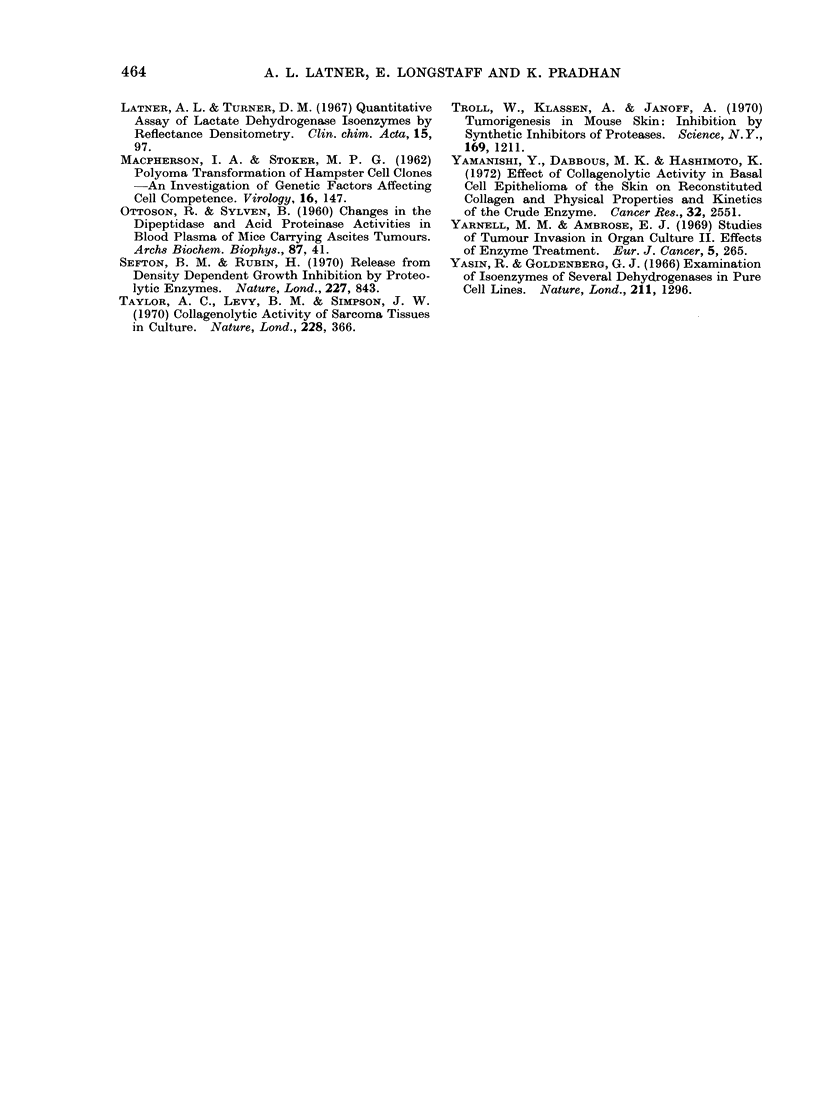# Inhibition of Malignant Cell Invasion in vitro by a Proteinase Inhibitor

**DOI:** 10.1038/bjc.1973.58

**Published:** 1973-06

**Authors:** A. L. Latner, E. Longstaff, K. Pradhan

## Abstract

The inhibitory effect of the protease inhibitor aprotinin (Trasylol) on the invasion of mouse kidney explants by polyoma virus transformed BHK21 cells was investigated using a mixed cell/organ culture technique. The extent of invasion was monitored by following the changes in LDH isoenzyme pattern in the explants and by histological assessment. The kidney explants containing aprotinin were found to maintain a normal kidney LDH pattern and to suffer considerably less invasion than the explants not containing the drug. These results support the idea that proteolytic enzymes are associated with invasion and that inhibitors of protease activity could possibly be useful in the management of clinical cancer.


*Br. J. Cancer* (1973) **27**, 460

### INHIBITION OF MALIGNANT CELL INVASION *IN VITRO* BY A PROTEINASE INHIBITOR

A. L. LATNER, E. LONGSTAFF AND K. PRADHAN*

*From the Cancer Research Unit, Department of Clinical Biochemistry, The Royal Victoria Infirmary, Newcastle upon Tyne, NE1 7RU, and the Department of Pathology,* Royal Berkshire Hospital, London Road, Reading*

Received 15 February 1973. Accepted 10 March 1973

**Summary.—The inhibitory effect of the protease inhibitor aprotinin (Trasylol) on the invasion of mouse kidney explants by polyoma virus transformed BHK21 cells was investigated using a mixed cell/organ culture technique. The extent of invasion was monitored by following the changes in LDH isoenzyme pattern in the explants and by histological assessment. The kidney explants containing aprotinin were found to maintain a normal kidney LDH pattern and to suffer considerably less invasion than the explants not containing the drug. These results support the idea that proteolytic enzymes are associated with invasion and that inhibitors of protease activity could possibly be useful in the management of clinical cancer.**

THE notion that the ability of malignant tumours to invade and destroy normal tissues may be associated with their capacity to produce proteolytic enzymes has been held for some time but firm experimental evidence to support this association has not yet been established. However, there is considerable evidence that proteolytic enzymes are present in animal neoplasms (Ottoson and Sylven, 1960) and that collagenolytic enzymes occur in many human tumours (Taylor, Levy and Simpson, 1970; Dresden, Heilman and Schmidt, 1972; Yamanishi, Dabbous and Hashimoto, 1972). Also, trypsin has been shown to encourage the invasion of malignant cells into foetal tissues in *in vitro* systems (Yarnell and Ambrose, 1969).

To test the hypothesis further, we decided to study the ability of malignant cells to invade tissue explants containing an inhibitor of proteolytic activity using an established *in vitro* system (Latner, Longstaff and Lunn, 1971).

### MATERIALS AND METHODS

The invasion of mouse (Bar Harbor strain 129) kidney cortex explants by polyoma virus transformed hamster kidney cells (BHK21/C13/Py, Macpherson and Stoker, 1962) was investigated using the mixed cell/organ culture technique developed by Latner *et al.* (1971). Monolayers of cells were grown in the filter wells of the apparatus and kidney explants cultured above them. After 7 days in culture, the kidney explants were removed and examined.

The broad spectrum protease inhibitor aprotinin (Trasylol, Bayer) was selected for study because it is known to accumulate rapidly in the renal tissues when administered intravenously (Haberland, 1967). We have verified by preliminary observations that aprotinin is an inhibitor of collagenase activity. This was done by measuring the proteolytic activity by the method of Goldspink, Holmes and Pennington (1971) of a purified specimen of collagenase (Sigma Chemical Co. Ltd) with bovine Achilles tendon collagen (Sigma Chemical Co. Ltd) as a substrate. In the presence of aprotinin, collagenase activity was undoubtedly inhibited.

The test kidney explants were primed with inhibitor by injecting mice, one hour before sacrifice, with 8000 kallikrein inactivating units (KIU) of aprotinin *via* the caudal vein.

The extent of invasion of the kidney explants by the Py cells was monitored initially in 2 experiments by comparing the percentage M sub-unit contribution to the lactate dehydrogenase isoenzyme (LDH) pattern of control and test cultures (calculated from each isoenzyme assuming tetrametic composition) but more recently by direct histological methods. It should be pointed out that LDH of Py cells consists solely of M units and that mouse kidney contains an appreciable proportion of H units. Consequently, an increase in the M sub-unit contribution to the host tissue's LDH pattern should be indicative of Py cell invasion. To test this supposition, 12 preliminary experiments were undertaken; in each, untreated mouse kidney was used for organ culture. The individual experiment consisted of a control and a test, each of which contained 8 mouse kidney explants. In the control group, the explants were cultured alone; in the test group they were cultured on top of a confluent layer of Py cells (Latner *et al.*, 1971).

The percentage M sub-unit contribution to the LDH isoenzyme patterns of the cultured explants was estimated quantitatively following vertical starch gel electrophoresis by reflectance densitometry according to the method of Latner and Turner (1967).

In experiments in which the LDH patterns were determined, half the number of explants in each filter well were selected at random and used for enzyme extraction. The remaining explants in these experiments were fixed in Carnoy's fluid, sectioned at 6 $\mu$m thickness, stained with haematoxylin and eosin and examined microscopically. The invading cells were readily recognized.

In 2 further experiments in which the LDH patterns were not determined, all the explants were used for quantitative histological assessment. In these studies the extent of invasion was estimated by preparing serial sections of the explants and projecting the image of every tenth section on to Whatman chromatography paper grade 3MM, drawing round the whole section and its invaded area and weighing the cut out areas representing the whole sections and then those corresponding to invaded areas. The resulting numerical populations from control and test groups were analysed using the non-parametric statistical technique of Mann and Whitney as described by Campbell (1967).

In addition, the possible toxic effect of aprotinin on Py cells in monolayer culture was investigated by adding Eagle's E4 medium plus 20% calf serum (Flow Laboratories) and containing 500 KIU/ml aprotinin to growing cultures of cells and studying their morphology over a 3-day incubation period.

### RESULTS

The 12 preliminary paired experiments demonstrated that increases in the percentage M sub-unit composition of invaded explants could be detected consistently. The mean percentage M sub-unit composition of normal kidney cortex cultured in the absence of Py cells was found to be 42·5% with a standard deviation of $\pm 6{\cdot}9\%$. The corresponding mean values for invaded explants were found to be $51{\cdot}7 \pm 11{\cdot}5\%$. The increases were found to be statistically significant ($P < 0{\cdot}01$) and Py cell invasion was confirmed histologically in each trial. The relatively large " within group " variation in these estimations has been found to be due almost entirely to the inherent variation between electrophoresis gels. The variation in estimates of sub-unit composition of similar samples on the same electrophoresis gel (*i.e.* the " within gels " variation) has been shown to be relatively small (a standard deviation of less than 2% is readily obtainable) and consequently paired control and test estimations were always made on the same gel. In the two experiments concerned with the LDH estimations of aprotinin-primed explants, the 2 test and 2 control materials were all subjected to electrophoresis on the same gel. The changes in percentage M contribution that were detected when the explants were challenged by Py cells fell inside the expected " within gel " variation and relatively little invasion could be detected histologically. The mean values obtained for the percentage M sub-unit composition were 44·5% for the unchallenged aprotinin-primed explants and 43·5% for the challenged aprotinin-primed explants.

In the following 2 experiments, LDH was not estimated and all explanted tissues were assessed histologically. The results obtained from the micro-projection of the serial sections are presented in Table I. The mean area of explant invaded in the unprimed explants (controls) was found to be 5·96% whereas the aprotinin-primed explants (tests) were invaded on the average only 2·39%. Statistical analysis of the data revealed that there were significant reductions of invasion in the aprotinin-primed explants compared with the controls when the total weights of the invaded areas were considered ($P = 0{\cdot}04$), and also when the percentage invaded areas were considered ($P = 0{\cdot}004$). No significant difference was found between the total weights of sections of the explants of control and test cultures.

Aprotinin did not appear to be toxic to the Py cells in monolayer culture and the cells continued to grow to confluence in the same time as similar untreated control cultures. However, the treated cells appeared to be somewhat more spread out on the growth surface and remained more securely attached to the growth surface than the controls when subjected to mechanical agitation.

### DISCUSSION

The 2 polypeptide sub-units H and M of lactate dehydrogenase are combined in tetrads to form the active enzyme molecules. Normally, 5 electrophoretically distinct LDH isoenzymes can be distinguished in somatic tissues but frequently, in malignant tissues and cells in artificial culture, LDH-5 is produced in significantly

TABLE I.—*Comparison Between the Total Weights, Invaded Weights and Percentage Invasion, of Every Tenth Serial Section of Unprimed Kidney Explants (Controls) and Aprotinin-primed Explants (Tests)*

| | Controls | | | Tests | | |
|---|---|---|---|---|---|---|
| Explant | Total weight explant section areas (arb. units) | Total weight invaded areas (arb. units) | % explant areas invaded | Total weight explant section areas (arb. units) | Total weight invaded areas (arb. units) | % explant areas invaded |
| A | 27·69 | 1·76 | 6·35 | 11·71 | 0·38 | 3·25 |
| B | 23·58 | 0·17 | 0·72 | 12·67 | 0·11 | 0·87 |
| C | 25·04 | 0·29 | 1·16 | 28·34 | 1·71 | 6·03 |
| D | 24·23 | 0·93 | 3·84 | 21·13 | 0·24 | 1·14 |
| E | 13·47 | 0·84 | 6·24 | 8·43 | 0·00 | 0·00 |
| F | 24·85 | 1·52 | 6·12 | 24·94 | 0·50 | 2·00 |
| G | 22·67 | 1·61 | 7·10 | 12·06 | 0·00 | 0·00 |
| H | 11·26 | 0·04 | 0·36 | 23·22 | 2·44 | 10·51 |
| I | 14·60 | 0·30 | 2·05 | 15·74 | 0·00 | 0·00 |
| J | 11·16 | 0·83 | 7·44 | 12·66 | 0·20 | 1·58 |
| K | 35·70 | 5·80 | 16·25 | 36·52 | 0·42 | 1·15 |
| L | 23·94 | 3·41 | 14·25 | 38·43 | 0·26 | 0·68 |
| M | 11·34 | 1·88 | 16·58 | 24·43 | 0·42 | 1·72 |
| N | 15·73 | 0·30 | 1·91 | 37·85 | 0·21 | 0·55 |
| O | 9·25 | 0·64 | 6·92 | 17·98 | 0·37 | 2·06 |
| P | 26·16 | 1·75 | 6·69 | 53·25 | 0·15 | 0·30 |
| Q | 18·96 | 1·05 | 5·54 | 25·20 | 1·04 | 4·13 |
| R | 22·07 | 1·11 | 5·03 | 29·45 | 0·36 | 1·22 |
| S | 24·88 | 0·66 | 2·65 | 28·92 | 2·12 | 7·33 |
| T | 10·22 | 0·20 | 1·96 | 22·17 | 0·58 | 2·62 |
| U | — | — | — | 16·02 | 0·45 | 2·81 |
| Mean values | 19·84 | 1·26 | 5·96% | 23·86 | 0·57 | 2·39% |

increased amounts and polyoma-transformed BHK21 cells produce only LDH-5 (Yasin and Goldenberg, 1966). In theory, therefore, the invasion of Py cells into mouse kidney cortex could be monitored by estimating the percentage contribution of M sub-units in the explants, and we were able to demonstrate consistently a significant increase in percentage M sub-units in 12 trials involving Py cell invasion. Since there was no significant change in the percentage M sub-units in aprotinin-primed explants exposed to Py cells, nor any appreciable invasion observed, it follows that the protease inhibitor aprotinin inhibited the invasion of Py cells. Studies involving the microprojection of serial sections confirmed this view, since the data obtained from these investigations revealed that the inhibition of invasion in the aprotinin-primed explant was statistically significant.

Since aprotinin is a fairly broad spectrum protease inhibitor a precise definition of its mode of action is not yet available, but the results reported here demonstrate that invasion, in the *in vitro* system at least, can be significantly reduced by inhibition of proteolytic activity. Because aprotinin was not found to be toxic to BHK21/Py cells in monolayer culture, it cannot be argued that the inhibition of invasion of the aprotinin-primed kidney explants was due primarily to the toxicity of the inhibitor, but rather that the invading cells depended upon the action of proteases.

It is interesting to note that the addition of low concentrations of trypsin to confluent cultures of normal chick embryo cells has been shown to release them from density dependent growth inhibition (Sefton and Rubin, 1970). Cell division and escape from contact inhibition of growth has been demonstrated when certain proteolytic enzymes were added to confluent cultures of non-malignant mouse fibroblasts in amounts too small to produce detachment (Burger, 1970). Conversely, inhibitors of proteolytic activity have been found to inhibit promotion by croton oil or phorbol ester of tumorigenesis in mouse skin initiated by dimethylbenzanthracene (Troll, Klassen and Janoff, 1970). Treatment with phorbol ester resulted in an increase in protease activity in the skin. Similar results have been reported by Hozumi *et al.* (1972) using the protease inhibitor leupeptin. Protease inhibitors have also been found to promote parallel alignment of hamster tumour cells in culture, to increase the adhesiveness of rounded cells and to depress cellular proliferation (Goetz, Weinstein and Roberts, 1972).

Bearing all this information in mind, it would seem reasonable to postulate that one of the biochemical prerequisities of an invasive tumour could be the ability to secrete proteolytic enzymes which could break down the intercellular matrix of the host tissues and facilitate the mechanical invasion of the tumour cells as well as aid in their supply of nutrient.

Whatever the mechanisms are that are involved, the results reported here could indicate the possible effectiveness of protease inhibitors in the chemotherapy of invasive tumours.

### REFERENCES

BURGER, M. M. (1970) Proteolytic Enzymes Initiating Cell Division and Escape from Contact Inhibition of Growth. *Nature, Lond.*, **227**, 170.

CAMPBELL, R. C. (1967) *Statistics for Biologists.* London: Cambridge University Press.

DRESDEN, M. H., HEILMAN, S. A. & SCHMIDT, J. D. (1972) Collagenolytic Enzymes in Human Neoplasms. *Cancer Res.*, **32**, 993.

GOETZ, I. E., WEINSTEIN, C. & ROBERTS, E. (1972) Effects of Protease Inhibitors on Growth of Hamster Tumour Cells in Culture. *Cancer Res.*, **32**, 2469.

GOLDSPINK, D. F., HOLMES, D. & PENNINGTON, R. J. (1971) Studies of Proteolytic Activity in Commercial Myoglobin Preparations. *Biochem. J.*, **125**, 865.

HABERLAND, G. L. (1967) *Biochemistry of Trasylol.* Proceedings of a Symposium on Proteinase Inhibition in Medicine and Surgery. Royal Society of Medicine, London.

HOZUMI, M., OGAWA, M., SUGIMURA, T., TAKEUCHI, T. & UMEZAWA, H. (1972) Inhibition of Tumorigenesis in Mouse Skin by Leupeptin, A Protease Inhibitor from *Actinomycetes.* *Cancer Res.*, **32**, 1725.

LATNER, A. L., LONGSTAFF, E. & LUNN, J. M. (1971) Invasive Properties of Histone Transformed Cells. *Br. J. Cancer.*, **25**, 568.

LATNER, A. L. & TURNER, D. M. (1967) Quantitative Assay of Lactate Dehydrogenase Isoenzymes by Reflectance Densitometry. *Clin. chim. Acta*, **15**, 97.

MACPHERSON, I. A. & STOKER, M. P. G. (1962) Polyoma Transformation of Hampster Cell Clones —An Investigation of Genetic Factors Affecting Cell Competence. *Virology*, **16**, 147.

OTTOSON, R. & SYLVEN, B. (1960) Changes in the Dipeptidase and Acid Proteinase Activities in Blood Plasma of Mice Carrying Ascites Tumours. *Archs Biochem. Biophys.*, **87**, 41.

SEFTON, B. M. & RUBIN, H. (1970) Release from Density Dependent Growth Inhibition by Proteolytic Enzymes. *Nature, Lond.*, **227**, 843.

TAYLOR, A. C., LEVY, B. M. & SIMPSON, J. W. (1970) Collagenolytic Activity of Sarcoma Tissues in Culture. *Nature, Lond.*, **228**, 366.

TROLL, W., KLASSEN, A. & JANOFF, A. (1970) Tumorigenesis in Mouse Skin: Inhibition by Synthetic Inhibitors of Proteases. *Science, N.Y.*, **169**, 1211.

YAMANISHI, Y., DABBOUS, M. K. & HASHIMOTO, K. (1972) Effect of Collagenolytic Activity in Basal Cell Epithelioma of the Skin on Reconstituted Collagen and Physical Properties and Kinetics of the Crude Enzyme. *Cancer Res.*, **32**, 2551.

YARNELL, M. M. & AMBROSE, E. J. (1969) Studies of Tumour Invasion in Organ Culture II. Effects of Enzyme Treatment. *Eur. J. Cancer*, **5**, 265.

YASIN, R. & GOLDENBERG, G. J. (1966) Examination of Isoenzymes of Several Dehydrogenases in Pure Cell Lines. *Nature, Lond.*, **211**, 1296.